# Of mice and men: models and mechanisms of diabetic cardiomyopathy

**DOI:** 10.1007/s00395-018-0711-0

**Published:** 2018-11-15

**Authors:** Christian Riehle, Johann Bauersachs

**Affiliations:** Department of Cardiology and Angiology, Hannover Medical School, Carl-Neuberg-Str. 1, Hannover, 30625 Germany

**Keywords:** Heart failure, Diabetes mellitus, Diabetic cardiomyopathy, Cardiac energetics, Mitochondria, Animal models

## Abstract

Diabetes mellitus increases the risk of heart failure independent of co-existing hypertension and coronary artery disease. Although several molecular mechanisms for the development of diabetic cardiomyopathy have been identified, they are incompletely understood. The pathomechanisms are multifactorial and as a consequence, no causative treatment exists at this time to modulate or reverse the molecular changes contributing to accelerated cardiac dysfunction in diabetic patients. Numerous animal models have been generated, which serve as powerful tools to study the impact of type 1 and type 2 diabetes on the heart. Despite specific limitations of the models generated, they mimic various perturbations observed in the diabetic myocardium and continue to provide important mechanistic insight into the pathogenesis underlying diabetic cardiomyopathy. This article reviews recent studies in both diabetic patients and in these animal models, and discusses novel hypotheses to delineate the increased incidence of heart failure in diabetic patients.

## Introduction

The prevalence of diabetes is increasing at an alarming rate. Estimations by the World Health Organization (WHO) reported that 422 million adults were affected by diabetes in 2014, compared to 108 million in 1980. In 2004, 3.4 million people died due to complications associated with diabetes, and this number is expected to double by 2030 (www.who.int). There are two predominant types of diabetes; type 1 diabetes (T1D) is characterized by impaired insulin production and insulinopenia as a primary result of an autoimmune response against pancreatic β-cells. In contrast, hallmarks of type 2 diabetes (T2D) are peripheral insulin resistance and pancreatic β-cell failure during the later course of the disease [164].

Diabetes induces micro- and macroangiopathy. The main cause of morbidity and mortality in diabetic patients are cardiovascular complications [175]. Numerous clinical trials indicate diabetes as a major risk factor for myocardial infarction. Similarly, diabetes increases the risk of future heart failure up to fivefold [66, 104, 105, 109, 137]. However, the high prevalence of heart failure in diabetic patients is not explained by concomitant hypertension and coronary artery disease. Over four decades ago, Rubler and colleagues reported autopsy data from diabetic patients with left ventricular (LV) dilatation in the absence of any obvious aetiology for heart failure [169]. Later reports confirmed the initial observation [89, 161], resulting in the concept of “diabetic cardiomyopathy” (DCM). DCM is mainly characterized by diastolic dysfunction in the absence of systolic dysfunction (heart failure with preserved ejection fraction, HFpEF) [125] and increased fibrosis in the absence of hypertension or coronary artery disease. The pathomechanisms underlying the development of DCM are multifactorial and incompletely understood. Consequently, no treatment to prevent or reverse the underlying molecular changes exists at this time [133].

Diabetes has adverse effects on the different cell types of the heart, including endothelial cells [63], fibroblasts [170] and cardiomyocytes. Various small and large animal models of T1D and T2D have been generated to study the impact of diabetes on the heart. These models are based on genetic manipulations, dietary interventions, and treatment with pancreatic toxins, which mimic many aspects of diabetes and DCM. In the present review, we will focus on studies performed on diabetic patients and rodent models. We will explore mechanisms, which are mainly present in cardiomyocytes and underlie the pathogenesis of DCM. Before these mechanisms will be discussed in detail, a brief introduction of the most commonly used animal models of T1D and T2D including their strengths and limitations of their use is warranted.

## Animal models

Rodents, especially mice and rats, are powerful tools to study the mechanisms involved in the development of DCM. The human, mouse, and rat genomes have nearly the same size, each containing about 30,000 protein-coding genes, with about 99% of the genes encoded in the mouse genome having a homologue in humans [82, 142]. In addition to these genomic similarities, further advantages of rodent models are the short breeding cycle and the availability of a variety of genetically engineered gain-of-function and loss-of-function models. The main characteristics of commonly used rodent models to study various aspects of DCM in comparison with findings from T1D and T2D patients are summarized in the Table 1, and will be discussed in detail below.

**Table 1:** Characteristics of diabetic patients and selected animal models of type 1 and type 2 diabetes

	Type 1 diabetes					Type 2 diabetes							Additional transgenic mouse models	
	Patients	Animal models				Patients	Animal models							
		Pharmacological	Transgenic			Obesity/type 2 diabetes	Diet-induced ± low-dose STZ		Transgenic					
		STZ	OVE26	NOD	Akita		HFD/HSD	HFD + low-dose STZ	ob/ob	db/db	ZF/ZDF	GK	MHC-PPARα	CIRKO
Fatty acid oxidation	↑ [92, 93]	↑ [36, 95]			↑ [12, 34]	↑ [153]	↑ [27, 219]		↑ [32, 134]	↑ [2, 32, 95]	↑ [208]		↑ [73]	↓ [14]
Glucose oxidation	↓ [92, 93]	↓ [36, 95]			=/↓ [12, 34]	=/↓ [152, 153]	↓ [219]		↓ [32, 134]	↓ [32, 95]	↓ [83]		↓ [73]	↓ [26]
Cardiac efficiency		=/↓ [36, 95]			= [34]	↓ [153]	= [27]		↓ [28]	↓ [29, 95]	= [228]			
Mitochondrial function		↓ [36, 74, 95, 117]	↓ [179, 180]		↓ [34, 35]	↓ [5, 141]	= [27, 219]		↓ [28]	↓ [29]				↓ [26]
Mitochondrial content			↑ [179, 180]		↑ [34, 35]	= [141]			↑ [25]	↑ [29]				↑ [26]
Ca^2+^ dynamics		↓ [114, 190, 195, 230]	↓ [226]		↓ [116]	↓ [102]			↓ [65, 70, 120]	↓ [15, 151]		↓ [58]	↓ [73]	
Oxidative stress		↑ [7, 36, 117, 187]	↑ [179, 226]		= [34]	↑ [5, 6, 84, 141]	↑ [27, 193]	↑ [132, 144]	↑ [120]	↑ [29]	=/↑ [7, 54, 205]	↑ [172]	↑ [72]	↑ [26]
Triglycerides/lipotoxicity	= [90]	↑ [72]		↑ [64]	↑ [12, 156]	↑ [135, 145, 167, 177]	=/↑ [13, 27, 43, 158]		↑ [32, 48]	↑ [1, 32]	↑ [177, 232]		↑ [72]	= [14]
RAAS activation		↑ [30, 187]				↑ [77]								
Inflammation		↑ [196, 214–217]	↑ [210]	↑ [64]	↑ [38]		↑ [111, 139, 193]				↑ [100]			
AGE		↑ [7, 18, 19, 37, 114, 128, 148]				↑ [149]	↑ [193]			↑ [147]	↑ [7]			
ER stress		↑ [119, 124, 222]					↑ [123, 225]							
Autophagy		↓ [231]	↓ [221]			=/↑ [141, 143]	↓/=/↑ [91, 112, 136]							
Cell death	↑ [46]	↑ [22, 96, 103, 173]				↑ [6, 46, 77]			↑ [10]	↑ [10]	↑ [232]			
Fibrosis		↑ [37, 187, 203]	↑ [210]		= [12]	↑ [161, 182]	↑ [136, 158]		= [48, 202]		↑ [232]		= [73]	= [14]
Contractile function	↓/=/↑ [31, 47, 168, 174, 184, 229]	↓ [96, 114, 128, 195]	↓ [179, 210, 221, 226]	↓ [64, 150]	↓ [12, 34]	↓ [69, 75, 104, 105, 109]	=/↓ [13, 158, 198, 219]	↓ [132]	↓/=/↑ [32, 48, 134]	=/↓ [1, 29, 32, 147]	↓ [83, 177]	↓ [98]	↓ [73]	=/↓ [14, 26]
Cardiac size	=/↑ [47, 110, 168, 174]	↓ [36, 37, 95]	↑ [210]	↓ [150]	↓ [12, 34]	↑ [62]	=/↑ [43, 158, 193, 198]	↑ [132, 144]	=/↑ [28, 32, 48, 65, 120, 134]	= [1, 2, 29, 32]	↑ [54]	↑ [61]	↑ [73]	↓ [14]

↑ increased; ↓ decreased; = no difference observed. AGE, advanced glycation end products; CIRKO, mice with cardiomyocyte-selective insulin receptor deletion; ER, endoplasmic reticulum; GK, Goto-Kakizaki rats; HFD/HSD, high fat/high sucrose diet; MHC-PPARα, mice with cardiomyocyte-specific overexpression of peroxisome proliferator activated receptor α (PPARα); NOD, non-obese diabetic mice; OVE26, OVE26 diabetic mice; RAAS, renin–angiotensin–aldosterone system; STZ, streptozotocin; ZDF, Zucker diabetic fatty rats; ZF, Zucker fatty rats

Streptozotocin (STZ) is a glucosamine-nitrosourea compound, which is toxic to pancreatic β-cells. Following intraperitoneal injection, STZ is transported into pancreatic β-cells by the glucose transporter 2 (GLUT2) based on its structural similarity to glucose, which results in necrosis and subsequent loss of insulin production [23]. STZ models are used to study both T1D and T2D. High-dose STZ protocols are primarily used to study T1D. Owing to the low penetrance of T2D development with high fat diet (HFD) chow feeding, more recent models have taken advantage of the clinical presentation of late stage T2D and β-cell destruction by adding in very low dose of STZ [11, 132, 144, 160, 218]. Another model of T1D is the OVE26 mouse, which overexpresses the Ca^2+^-binding protein calmodulin in pancreatic β-cells, resulting in pancreatic β-cell damage. Non-obese diabetic (NOD) mice develop T1D as a result of leukocyte infiltrate of the pancreatic islets, causing insulitis, and β-cell failure [131]. T1D Akita mice (*Ins2*^Akita+/−^) exhibit a spontaneous mutation in the *Insulin2* gene, which facilitates misfolding of the insulin protein, endoplasmic reticulum (ER) stress and ultimately β-cell failure [227]. Although each of these models accurately reflects the insulin deficient nature of T1D, there are some limitations of which the most noteworthy is that they do not adequately capture the autoimmune contribution to the development of T1D in human patients [71, 155].

Commonly used transgenic models of obesity, insulin resistance and T2D are ob/ob [76] and db/db [40] mice, which are based on leptin deficiency or resistance, respectively. Similarly, Zucker fatty (ZF) rats develop obesity as a consequence of non-functional leptin receptors [154]. Zucker diabetic fatty (ZDF) rats were generated by inbreeding ZF rats with high serum glucose levels [51]. Goto-Kakizaki (GK) rats are an inbred strain derived from Wistar rats that spontaneously develop T2D [81]. Mice with adipose tissue-specific overexpression of sterol regulatory element-binding protein-1c (SREBP-1c) develop insulin resistance and elevated plasma triglyceride levels [183]. To avoid potential perturbations based on altered leptin concentrations and signalling, a variety of studies feed rodent models a HFD with increased caloric intake to induce obesity, insulin resistance, and T2D, which will be discussed in detail below.

**Fig. 1:**
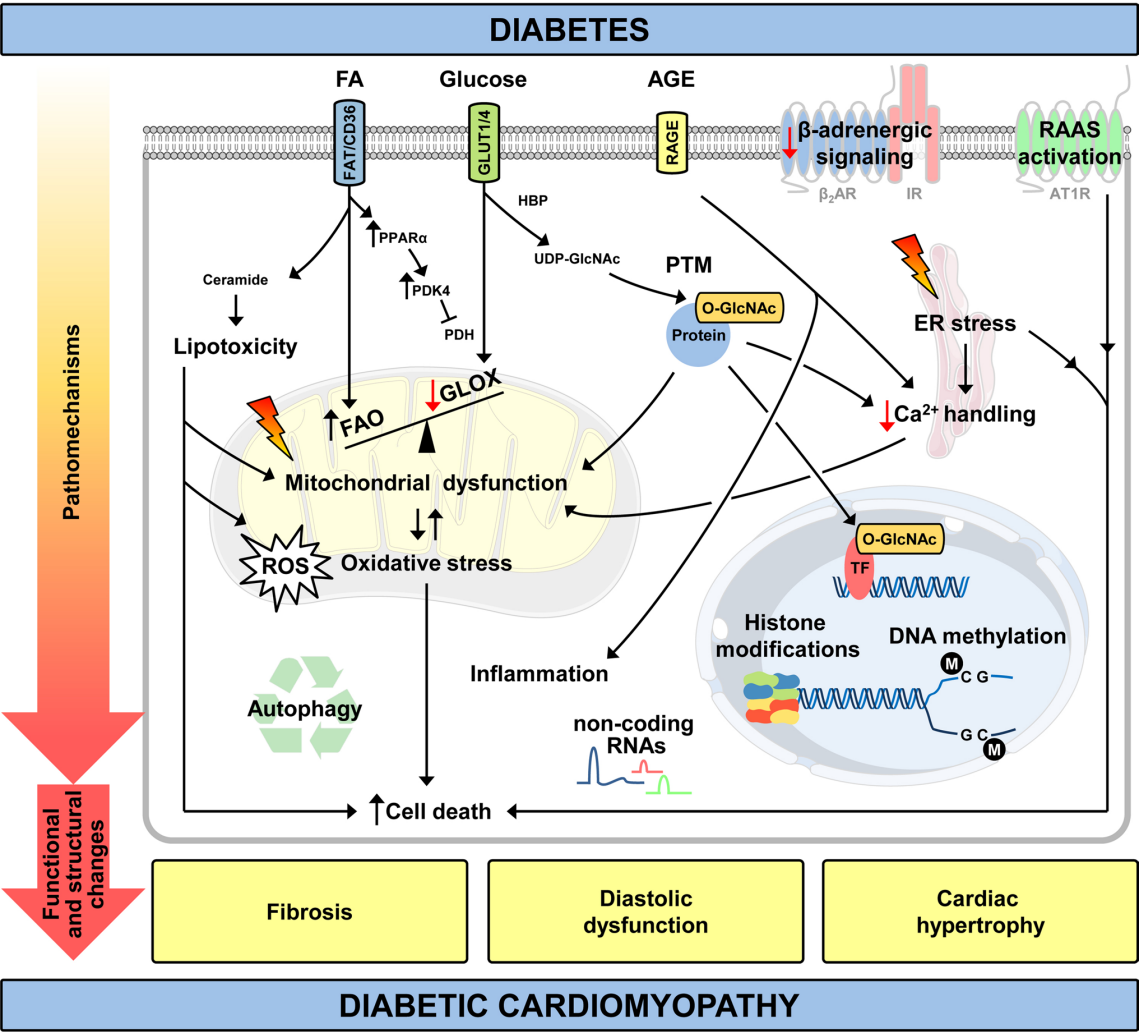
Pathomechanisms and clinical features of diabetic cardiomyopathy. ↑ increased/↓ decreased relative to normal conditions; AGE, advanced glycation end products; AT1R, angiotensin II receptor type 1; ER, endoplasmic reticulum; FAO, fatty acid (FA) oxidation; FAT/CD36, fatty acid translocase; GLOX, glucose oxidation; HBP, hexosamine biosynthetic pathway; IR, insulin receptor; PDH, pyruvate dehydrogenase; PDK4, pyruvate dehydrogenase kinase 4; PPARα, peroxisome proliferator activated receptor α; PTM, posttranslational modification; RAAS, renin–angiotensin–aldosterone system; RAGE, receptor for advanced glycation end products; ROS, reactive oxygen species; TF, transcription factor; UDP-GlcNAc, uridine diphosphate-*N*-acetylglucosamine; β_2_AR, β_2_-adrenergic receptor

In addition to these more direct models of diabetes, transgenic models that replicate aspects of DCM have been generated. For example, mice with cardiomyocyte-specific overexpression of the transcription factor peroxisome proliferator activated receptor α driven by the α myosin heavy chain gene promoter (MHC-PPARα) exhibit increased cardiac fatty acid oxidation (FAO) and a phenotype similar to DCM. The investigation of this models helps to explore mechanisms by which perturbed cardiac substrate oxidation impairs contractile function without systemic metabolic alterations that are associated with diabetes [72, 73]. Cardiomyocyte-selective insulin receptor knockout (CIRKO) mice are used to study the effect of decreased insulin signalling in cardiomyocytes without causing systemic metabolic disturbances [14]. The following sections and Fig. 1 summarize the main mechanisms that have been proposed to explain the increased risk of heart failure observed in T1D and T2D. The hypotheses generated are based on studies conducted on either animal models or diabetic patients.

### Animal studies with high caloric diets

Numerous studies use dietary treatments to induce obesity, insulin resistance, and T2D in rodents and large animal models [189]. The term “Western” diet is commonly used for diets with a high total fat and sucrose content, which allows mimicking pathologies that have been associated with the “Western” human dietary pattern. In contrast, rodent HFD chow typically contains a variable amount of fat and a variable amount of added cholesterol [94]. Importantly, rodents usually eat any kind of fruit or grain from plants when living in their natural habitat. “Western” diet and HFD chow, which is typically used for laboratory experiments, may contain a fat content of up to 60%. In comparison, the increase in fat intake in rodent models is proportionally higher compared to humans consuming “Western” diets.

HFD feeding with a relatively low fat content (45% calories from fat) is not associated with contractile dysfunction following a short feeding duration in mice; however, systolic dysfunction develops after a prolonged duration of 20 weeks [198]. In contrast, HFD feeding of mice with 60% fat content contributes to systolic dysfunction after only 10 weeks of feeding and increases mortality [13], which suggests potential toxic effects for high caloric diets with a relatively high fat content. Similarly, exposure to “Western” diet (36% fat content and 36% sugar content) leads to solely diastolic dysfunction, while systolic function is preserved after a total duration of 8 months [158]. Other important parameters that could provide an explanation for the different phenotypes observed are the duration of the dietary treatment and the genetic background of the species used [211].

An additional mechanism is a potential biphasic response of cardiac insulin signalling, even though not directly proven across these studies. Cardiac insulin signalling is preserved in T2D humans and rodent models following short-term HFD feeding [55, 219]. However, prolonged HFD feeding in animal models impairs Akt activation and forkhead box O-1 (FOXO1) transcription factor phosphorylation [13], which results in persistent FOXO1 nuclear localization and activation. The FOXO1-mediated adverse effects are multifactorial, including induction of autophagy, atrophy, and MHC isoform switching. The adverse consequences of persistent FOXO1 activation are supported by attenuated systolic dysfunction following long-term HFD feeding of mice with genetic deletion of FOXO1 [13]. Further experimental evidence is provided by transgenic animals with cardiomyocyte-specific deletion of IRS1 and IRS2, which exhibit severe heart failure [157, 166]. This effect is ameliorated by the deletion of FOXO1 [157]. The potential biphasic response of insulin signalling is important to consider in the design of future studies. Posttranslational modification of FoxO1 also mediates cardiac collagen and protein metabolism as reported in the context of ischemic heart failure [107].

Several studies subjected rodents to high caloric diets in addition to low-dose STZ treatment [11, 132, 144, 160, 218] to induce ß-cell dysfunction and insulinopenia, which are long-term complications of T2D. To objective of these studies is to overcome the potential low penetrance of diabetes development following HFD feeding in rodents. Similar to studies using HFD, the additional treatment with STZ promoted oxidative stress, cardiac hypertrophy and contractile dysfunction in diabetic mice [132, 144].

## Mechanisms contributing to myocardial dysfunction in diabetic patients and rodent models of T1D and T2D

### Altered substrate metabolism

**Fig. 2:**
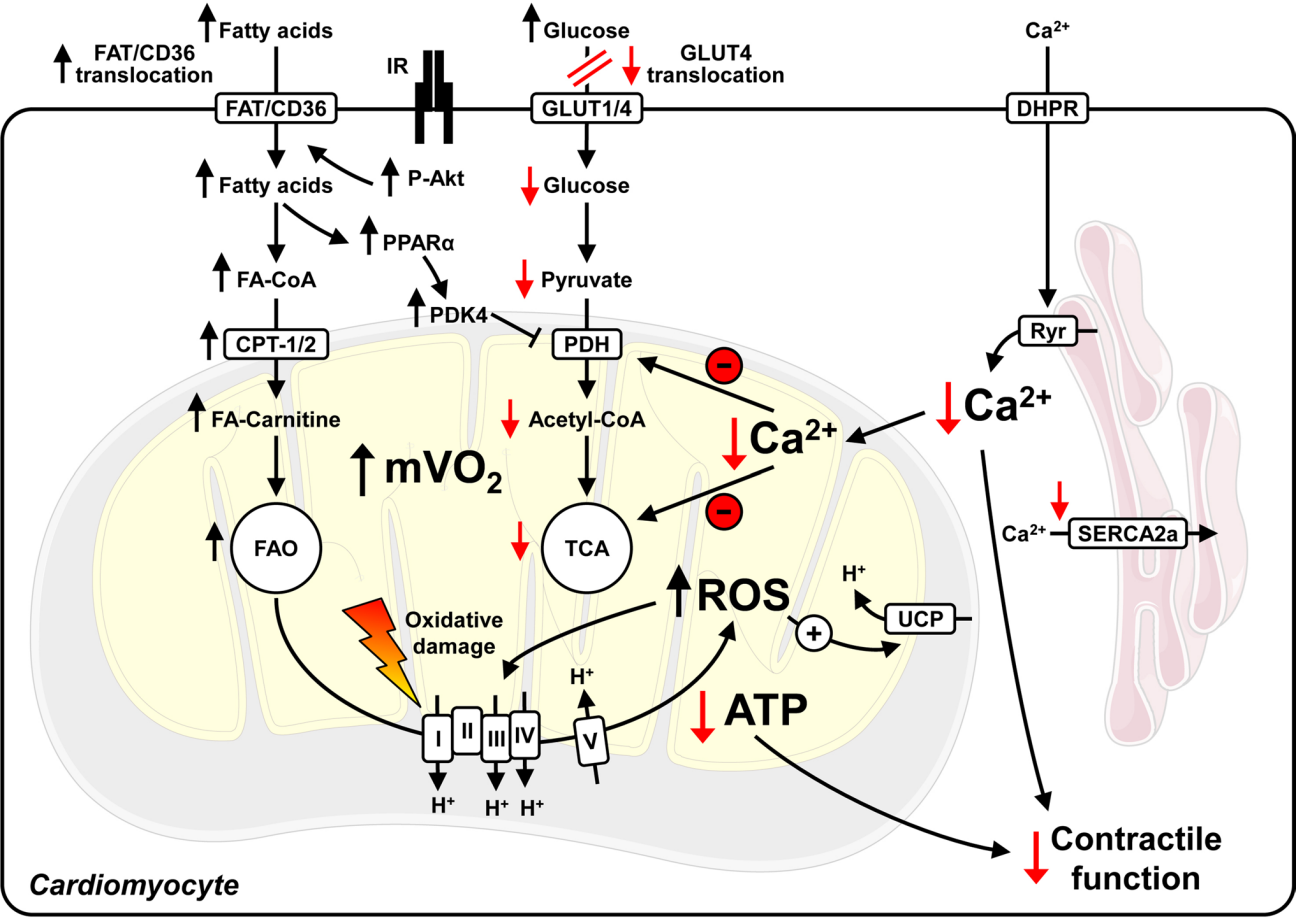
Mitochondrial uncoupling and perturbed Ca^2+^ dynamics in cardiomyocytes of type 2 diabetic hearts. Hyperinsulinemia activates insulin receptors (IR) and Akt, contributing to increased fatty acid translocase (FAT/CD36) transport to the plasma membrane, increased fatty acid (FA) uptake and fatty acid oxidation (FAO). Impaired GLUT4 expression and translocation attenuate glucose uptake and utilization, which further increases FAO and myocardial oxygen consumption (mVO_2_). Increased FAO stimulates the generation of reactive oxygen species (ROS), which may induce damage to proteins involved in oxidative phosphorylation and may activate uncoupling proteins (UCPs). Increased mitochondrial uncoupling enhances mVO_2_ and FAO, and decreases mitochondrial ATP production. As the increase in mVO_2_ is not paralleled by increased ATP production and contractility, cardiac efficiency (cardiac work/mVO_2_) decreases. Perturbed intracellular Ca^2+^ handling (reduced sarcoplasmic reticulum Ca^2+^ release by ryanodine receptors (Ryr) and impaired re-uptake by SERCA2a) reduce peak cytosolic Ca^2+^ levels, which may further decrease contractility and intramitochondrial Ca^2+^ levels. This limits the activity of mitochondrial enzymes and further compromises contractile function. Note that mitochondrial uncoupling, increased ROS and decreased cardiac efficiency are not observed in rodent models of type 1 diabetes. I–V, mitochondrial electron transport chain complexes I–V; CoA, Coenzyme A; CPT, carnitine palmitoyltransferase; DHPR, dihydropyridine receptor; PDK4, pyruvate dehydrogenase kinase 4; PPARα, peroxisome proliferator activated receptor α; TCA, tricarboxylic acid cycle. ↑ increased/↓ decreased relative to normal conditions

Diabetes is characterized by increased FAO and decreased glucose oxidation (GLOX, Figs. 1, 2) in the heart as described for T1D [92, 93] and T2D patients [152, 153], and several rodent models (summarized in the Table 1). Multiple mechanisms mediate the shift in substrate oxidation. The earliest defects are impaired translocation and abundance of glucose transporter 4 (GLUT4), as observed in a rodent model of HFD-induced obesity and insulin resistance [219]. These data imply impaired myocardial glucose utilization for the initial increase in FAO (Randle phenomenon [159]), even before any change in serum concentrations of free fatty acids and triglycerides. Glucose uptake and cellular membrane GLUT4 expression are decreased in heart tissue from T2D patients, while insulin receptor (IR) mediated signalling is increased [55]. An independent mechanism, which may increase fatty acid uptake in diabetic hearts, is enhanced fatty acid translocase (FAT/CD36) transport to the plasma membrane [57]. Under diabetic conditions, increased circulating concentrations of fatty acids increase the activity of the transcription factor PPARα [24]. PPARα drives the expression of genes involved in fatty acid uptake, transport, and oxidation [165]. In addition, PPARα induces the expression of pyruvate dehydrogenase kinase 4 (PDK4), thereby decreasing pyruvate dehydrogenase (PDH) activity and further suppressing GLOX [32, 219]. Importantly, hearts from MHC-PPARα mice are characterized by increased FAO and a metabolic phenotype similar to that found in DCM [73, 87].

ATP production from fatty acid substrates is less efficient than glucose-based ATP production (ATP generated/O_2_ consumed). Increased FAO rates in diabetic hearts enhance myocardial oxygen consumption (mVO_2_). However, contractile function does not increase and cardiac efficiency (cardiac work/mVO_2_) decreases. Studies in ob/ob and db/db mice indicate that mitochondrial uncoupling and increased reactive oxygen species (ROS) levels parallel the increase in FAO [28, 29]. The increase in ROS might be a consequence of an imbalance between a dysfunction of the mitochondrial electron transport chain and the increased amount of reducing equivalents generated by increased FAO. ROS have a very short half-life and are considered to cause cellular damage in close proximity to their origin, which implies mitochondria as a primary target of ROS [33]. ROS activate uncoupling proteins (UCPs) [67] that enable protons to bypass the ATP synthase embedded in the inner mitochondrial membrane, resulting in mitochondrial uncoupling (decreased coupling of mitochondrial ATP production to mVO_2_). Subsequently, FAO increases and cardiac efficiency is further impaired. Similar to T2D animal models, obese young women exhibit insulin resistance, increased mVO_2_ and FAO [153]. Importantly, fatty acid-mediated mitochondrial uncoupling, increased ROS levels and decreased cardiac efficiency are not present in T1D Akita mice [34], which is in contrast to T2D ob/ob and db/db mice [29, 120]. Thus, varied mechanisms might be responsible for the altered myocardial substrate utilization in the different types of diabetes. The hypothesis has been raised that ROS-mediated mitochondrial uncoupling may not be attributable to hyperglycaemia alone and may be a potential consequence of insulin resistance and T2D. This is supported by recent studies in CIRKO hearts, which exhibit increased oxidative stress and mitochondrial uncoupling under normoglycaemic conditions [26].

### Mitochondrial dysfunction

Mitochondrial dysfunction is a key feature of DCM and is observed in cardiac tissue from diabetic patients and models of T1D and T2D (see Table 1). Based on the mechanistic insight gained from rodent studies, the mechanisms for decreased mitochondrial oxidative capacity [5, 26–29, 34–36, 74, 95, 117, 179, 180, 219] include altered mitochondrial ultrastructure [25, 26, 29, 179, 180], proteomic remodelling [35, 88, 180, 200], and oxidative damage of proteins and mitochondrial DNA [206]. Additional mechanisms for mitochondrial dysfunction comprise perturbed mitochondrial Ca^2+^ dynamics, mitochondrial uncoupling in T2D and decreased cardiac insulin signalling in T1D, which are described in detail in the corresponding sections of this review. Compelling data for mitochondrial dysfunction in T2D patients have been provided by a series of studies by Anderson and colleagues. These studies utilized right atrial cardiac tissue, which exhibit mitochondrial dysfunction, increased oxidative stress, increased H_2_O_2_ emission, and increased sensitivity to Ca^2+^-induced opening of the mitochondrial permeability transition pore (mPTP) [5, 6]. Recently, Jelenik and colleagues showed decreased mitochondrial coupling and efficiency in ventricular tissue from patients with impaired insulin sensitivity [101]. Importantly, mitochondrial capacity is greater in ventricular compared to atrial tissue samples in humans, which provides a rationale to study mitochondrial function preferably in ventricular tissue [118]. Maximum respiration capacity is impaired in isolated mitochondria from patients with non-alcoholic steatohepatitis (NASH) and hepatic insulin resistance. This provides evidence for mitochondrial dysfunction under conditions of insulin resistance even before the onset of diabetes [113].

### Impaired Ca^2+^ handling

Ca^2+^ enters cardiomyocytes through voltage-dependent L-type Ca^2+^ channels (dihydropyridine receptor, DHPR) contributing to Ca^2+^ release from the sarcoplasmic reticulum by ryanodine receptors (Ryr) and contraction in systole. Intracellular Ca^2+^ concentrations decrease to diastolic levels following Ca^2+^ transport into the sarcoplasmic reticulum via SERCA2a and into the extracellular environment via the sarcolemmal Na^+^/Ca^2+^ exchanger (NCX). In addition to these widely studied mechanisms of Ca^2+^ handling more recent evidence supports that regulation of store-operated Ca^2+^ entry (SOCE) may also be important in the development of DCM, specifically via post-translational regulation of stromal interaction molecule 1 (STIM1) [233]. Intramitochondrial Ca^2+^ concentrations change during the contraction cycle [99], which promotes the activity of mitochondrial enzymes, i.e. PDH, isocitrate dehydrogenase and α-ketoglutarate dehydrogenase [60, 146]. Ca^2+^ handling is perturbed in T1D animal models; for example, following STZ treatment [190, 195, 230]. In T2D db/db mice, sarcoplasmic reticulum Ca^2+^ load is decreased, Ca^2+^ leakage from the sarcoplasmic reticulum is increased, and rates of Ca^2+^ decay are reduced [15, 151]. Similarly, ob/ob mice exhibit impaired mitochondrial Ca^2+^ handling and decreased rates of intracellular Ca^2+^ release following electrical stimulation [65, 70]. Cardiac fibres from T2D patients show decreased myofilament function as a consequence of impaired Ca^2+^ sensitivity and support the findings from rodent models [102]. Together, these studies indicate that perturbed Ca^2+^ handling accelerates the development of contractile dysfunction in T1D and T2D (Figs. 1 and 2).

### Oxidative stress

Oxidative stress plays an essential role in the development of DCM, as described for humans [5, 6, 84, 141] and rodent models of T1D and T2D (summarized in the Table 1). Oxidative stress can result from increased levels of ROS, which is caused by either increased mitochondrial ROS generation or decreased efficiency of ROS scavengers, i.e. glutathione peroxidase (GPX), catalase, and manganese superoxide dismutase (MnSOD). Oxidative stress regulates several adverse mechanisms, including protein oxidation, generation of lipid peroxides, and formation of reactive nitrogen species from nitric oxide, which contributes to intracellular nitrosylation, such as protein tyrosine nitration [199]. Evidence for increased oxidative stress is provided by a study that utilized cardiac tissue from T2D patients, in which increased emission of mitochondrial H_2_O_2_ and increased abundance of 3-nitrotyrosine- and 4-hydroxynonenal (HNE)-modified proteins were observed [6]. Furthermore, overexpression of MnSOD or catalase attenuates the onset of mitochondrial dysfunction and impaired cardiomyocyte contractility in T1D OVE26 mice [179, 226].

### Lipotoxicity

The uptake of lipid intermediates is increased in DCM. Intracardiomyocellular accumulation of toxic lipid metabolites accelerates myocyte death and contractile dysfunction. This is supported by several studies that utilized transgenic mouse models with increased cardiomyocyte fatty acid uptake, i.e. overexpression of fatty acid transport protein [44], long-chain acyl-CoA synthetase 1 (ACS) [45] and lipoprotein lipase with a cell-attachment glycosylphosphatidylinositol anchor, which precipitates lipotoxic cardiomyopathy, even in the absence of diabetes [224]. The proposed mechanisms have been reviewed in detail recently [213], and include increased ROS generation, changes in the ER membrane composition resulting in ER stress, increased apoptosis as a consequence of increased de novo ceramide biosynthesis, and remodelling of the mitochondrial membrane. A recent study using ACS transgenic mice revealed a novel mechanism for lipotoxic cardiomyopathy, in which posttranslational modifications of mitochondrial fusion and fission proteins increase mitochondrial fission [197]. Importantly, while numerous studies describe cardiac accumulation of triglycerides in diabetic patients [135, 145, 167, 177] and genetic rodent models of diabetes [1, 32, 48, 177, 232], triglycerides represent a marker for toxic lipid metabolite accumulation and lipotoxicity, as opposed to directly causing harmful effects [213].

### Renin–angiotensin–aldosterone system (RAAS) activation

RAAS hyperactivation contributes to cardiac remodelling. RAAS inhibitors (angiotensin-converting enzyme (ACE) inhibitors, angiotensin (AT) receptor blockers and aldosterone receptor antagonists) are well-established standard treatments for chronic heart failure. The activity of the RAAS is increased under diabetic conditions [53]. Similarly, in vitro studies in neonatal rat ventricular myocytes (NRVM) identified high glucose levels as stimulators for intracellular AT II synthesis [186]. AT II receptor type 1 (AT1R) density and synthesis are increased in T1D hearts, and the increase in fibrosis is partially inhibited following treatment with ACE inhibitors and AT receptor blockers [30, 187, 214]. Together, these studies suggest that RAAS activation adversely affects cardiac structure in DCM.

### Inflammation

Studies using rodent models of T1D and T2D identified a critical role for increased myocardial inflammation in the progression of DCM. Hearts from T1D mice and rats show increased leukocyte infiltration, increased levels of pro-inflammatory cytokines (TNFα and IL-1β), increased expression of vascular cell adhesion molecule-1 and intercellular adhesion molecule-1, and decreased activity of the collagen degrading matrix metalloproteinase (MMP), which increases inflammation and fibrosis [196, 214, 217]. Similar data were obtained from HFD-fed and T2D rodents [100, 111, 139]. The detrimental effects caused by increased inflammation are further supported by the beneficial outcomes of a variety of interventions, which decrease inflammation in the hearts of diabetic rodents, i.e. TNFα antagonism [217], transgenic activation of the kallikrein–kinin system [196], AT receptor antagonist treatment [214], and pharmacological inhibition of p38 MAPK [215] or interleukin converting enzyme [216].

### Advanced glycation end products (AGE)

Under hyperglycaemic conditions, AGE are formed both intra- and extracellularly via the Maillard reaction. AGE are a heterogeneous group of compounds that are formed following non-enzymatic binding of sugar derivatives to proteins, lipids and nucleic acids, which impairs the physiological function of the molecules bound [21, 185]. For example, AGE are formed on SERCA2a and Ryr, which perturbs Ca^2+^ dynamics [18, 19]. Furthermore, AGE crosslink collagen molecules, contributing to increased fibrosis and contractile dysfunction [148]. In addition, AGE bind to their cognate receptor, receptor for advanced glycation end products (RAGE), which is located on the cellular membrane. One mechanism for RAGE-mediated heart failure is activation of NF-κB signalling, which increases β-MHC expression, as evidenced by attenuated contractile dysfunction and β-MHC expression in db/db mice following blockage of RAGE signalling [147]. Similar effects are present in T1D and T2D rats following treatment with the antioxidant dehydroepiandrosterone (DHEA), thus indicating a critical role for oxidative stress in the activation of RAGE-mediated pathways under diabetic conditions [7]. Additional RAGE-mediated mechanisms are increased ROS production and pro-inflammatory signalling [21]. Transgenic overexpression of the methylglyoxal-metabolizing enzyme glyoxalase-1 (GLO1) in mice decreases methylglyoxal-AGE levels and attenuates the onset of heart failure following myocardial infarction. These mice exhibit increased vascular density and decreased cardiomyocyte apoptosis compared to wild-type controls, which is paralleled by increased recruitment of c-kit^+^ progenitor cells and their incorporation into the vasculature [20]. Repeated percutaneous infusions of cardiac mesenchymal cells in mice with ischemic cardiomyopathy significantly improve contractile function compared to a single dose treatment, which suggests that multiple infusions are required for the full therapeutic potential of cell therapy [86]. In STZ-induced T1D rats, treatment with the crosslink breaker ALT-711 decreases cardiac AGE levels, restores collagen solubility, and diminishes diabetes-induced gene expression [37]. Similarly, siRNA-mediated knockdown of RAGE attenuates LV dysfunction in T1D mice [128].

### ER stress

The main physiological function of the ER is Ca^2+^ storage and folding of proteins. Accumulation of unfolded proteins inside the ER lumen causes a stress response, termed ER stress, which can result in apoptotic cell death in rodent models of T1D and T2D [115, 222]. ER stress also activates the unfolded protein response (UPR), which attenuates this effect. The main task of the UPR is to maintain cellular integrity by decreasing protein synthesis, degrading misfolded proteins, and increasing the synthesis of chaperones, which facilitate protein folding. Numerous studies have suggested a causative role for oxidative stress in the induction of ER stress under diabetic conditions [119, 124, 222].

### Autophagy

Autophagy is an evolutionarily conserved process that recycles long-lived proteins and organelles to maintain cellular homeostasis. Depending on the extent of autophagy and its duration, autophagy can have both beneficial and detrimental effects. Perturbed autophagy is associated with the pathogenesis of infectious diseases, cancer, obesity, and various disease conditions of the heart, including ischemia/reperfusion injury, cardiac hypertrophy and DCM [112, 163]. Studies have provided opposing results in the context of DCM.

Autophagy is decreased in rodent models of T1D [221, 231]. The proposed mechanisms comprise repression of AMPK and activation of mTOR under hyperglycaemic conditions. In contrast, autophagy is increased in some animal models of diet-induced obesity and T2D, but the evidence for this has not been consistent [91, 112, 136]. The underlying mechanisms for the differences in autophagy in T1D relative to T2D and the various models investigated need further investigation.

While T1D is associated with insulinopenia and impaired cardiac insulin signalling, proximal insulin signalling is preserved in T2D [55, 219]. Preserved insulin/mTOR signalling could be predicted to suppress autophagy in T2D, which is in contrast to some of the prior investigations. Therefore, differences in insulin signalling cannot fully explain the differences in autophagy when comparing the different types of diabetes. This also suggests that multiple mechanisms regulate autophagy in DCM that are, at least in part, independent of cardiac insulin signalling. It is also important to note that autophagy is a highly dynamic process and the differences detected might be attributed to experimental limitations in determining autophagic flux. Thus, additional research is warranted to gain further mechanistic insight and elucidate the impact of autophagy in DCM.

### Posttranslational modification (PTM)

PTMs can alter the activity of proteins. Metabolic-driven PTMs are particularly important in diabetes, i.e. acetylation and O-GlcNAcylation. Sirtuins (SIRTs) are defined as NAD^+^-dependent class III histone deacetylases that deacetylate target proteins involved in FAO, glucose metabolism, and mitochondrial energetics. SIRTs are differentially regulated in models of heart failure and in animal models of T1D and T2D. For example, cardiac SIRT3 expression is decreased in HFD-fed mice, which increases acetylation of mitochondrial β-oxidation enzymes and increases FAO [4]. Expression of SIRT isoforms is mediated by dietary interventions and pharmacological treatment; for example, treatment with the anti-oxidant resveratrol increases the expression of SIRT1 [207], implying SIRTs as potential pharmacological targets.

Increased protein O-GlcNAcylation has adverse effects in DCM as recently reviewed [212]. Glucose is converted to fructose-6-phosphate in the first steps of glycolysis, which enters the hexosamine biosynthesis pathway (HBP). Under physiological conditions, about 5% of total glucose is metabolized in the HBP, which is further increased under diabetic conditions. Multiple pathways provide intermediates for the HBP, including metabolic pathways for the biosynthesis and degradation of amino acids, fatty acids, and nucleotides, which directly links the availability of nutrients to the substrate supply of the HBP. The end product of the HPB, uridine diphosphate-N-acetylglucosamine (UDP-GlcNAc), is transferred to serine or threonine residues of target proteins by the enzyme O-GlcNAc transferase (OGT), a process termed O-GlcNAcylation. In contrast to non-enzymatic AGE formation, O-GlcNAcylation is a reversible posttranslational modification, with UDP-GlcNAc removal catalysed by O-GlcNAcase (OGA). Multiple nuclear, cytoplasmic, and mitochondrial proteins are targets for O-GlcNAc modification. O-GlcNAcylation also plays a central role in Ca^2+^ homeostasis, as evidenced by the modification of transcription factors regulating the expression of SERCA2a [52] and the sarcoplasmic reticulum protein STIM1, thereby attenuating SOCE and Ca^2+^ signalling [233]. O-GlcNAcylation of Ca^2+^/calmodulin-dependent protein kinase 2 (CAMKII) impairs Ca^2+^ handling and increases the risk of cardiac arrhythmia in diabetes [68].

O-GlcNAcylation directly impairs mitochondrial capacity. Proteomic studies identified 86 mitochondrial proteins as O-GlcNAc targets, with target proteins involved in major metabolic pathways, including the FAO and tricarboxylic acid (TCA) cycles [129]. UDP-GlcNAc is transported from the cytosol into mitochondria by the pyrimidine nucleotide carrier (PNC1) and cardiac mitochondria express both OGA and OGT [9]. OGT expression is increased in mitochondria from T1D rat hearts and modulation of OGT or OGA activity affects mitochondrial capacity [9]. These data indicate that cardiac mitochondria express the required machinery for O-GlcNAc modification which, in turn, regulates mitochondrial capacity. Together, O-GlcNAcylation provides an exciting new area of research, linking the availability of nutrients to cardiac energetics and contractile dysfunction, which may accelerate the onset of heart failure in diabetes.

### Epigenetics

Epigenetics is a rapidly expanding area of research and refer to a heritable modification of gene expression without alterations in DNA sequences. The modifications include non-coding RNAs (i.e. microRNAs and long-noncoding RNAs), DNA methylation, and histone modifications. Epigenetics are important during embryogenesis and play a central role during development and the pathogenesis of various disease conditions, including DCM. In addition to the transgenerational nature of epigenetics, these modifications can also be part of transcriptional regulation and may be regulated by altered metabolic flux directly associated with hyperglycaemia and other metabolic changes seen in diabetes [56].

#### miRNAs and lncRNAs

MicroRNAs (miRNAs or miRs) are short, single-stranded, non-coding RNA molecules consisting of about 22 nucleotides. The majority of miRNAs are encoded within the introns of protein-coding and non-coding genes. miRNAs are evolutionarily conserved and regulate gene expression at the post-transcriptional level. The mechanisms of miRNA-based gene regulation include binding of miRNAs to mRNAs for later degradation or repression of translation. Each miRNA can target multiple mRNAs, which provides the possibility to a single miRNA to orchestrate an entire pattern of gene expression. miRNAs play an important role in the regulation of cellular energy homeostasis, metabolism, and pathogenesis of numerous diseases, including diabetes. For example, miRNAs-103/107 are up-regulated in livers from ob/ob and diet-induced obese mice, and silencing of miRNAs-103/107 improves glucose homeostasis and insulin sensitivity [194]. Numerous miRNAs regulate cardiac fibrosis and hypertrophy [191]. For example, miRNA-21 augments pathological cardiac remodelling by stimulating MAP kinase signalling in fibroblasts [192]. Similarly, miRNAs are differentially regulated in diabetic hearts [85, 126, 178]. One example is miRNA-223, which is upregulated in LV biopsies from T2D patients and regulates GLUT4 expression and glucose uptake [126]. As previously reviewed, miRNA expression is altered in rodent models of T1D and T2D [85, 176]. One example of this has been observed in T2D ZDF rats, in which dysregulated miRNA-29 expression is correlated with cardiac structural damage [8]. Furthermore, differences in miRNA profiles regulate the hyperglycaemic memory in DCM. miRNA array analysis performed on LV tissue from STZ-induced T1D mice indicated dysregulation of 316 out of 1008 miRNAs. Following normalization of blood glucose levels by insulin treatment, the expression of 268 miRNAs remained significantly altered, thus suggesting a contribution of miRNAs to glycaemic memory. Ingenuity pathway analysis indicates that dysregulated miRNAs are implicated in myocardial signalling networks regulating autophagy, hypertrophic growth, oxidative stress, fibrosis, and heart failure, all of which are characteristics of DCM [39].

Long-noncoding RNAs (lncRNAs) are transcripts that are longer than 200 nucleotides, which can repress or enhance gene expression [204]. Similarly to miRNAs, lncRNAs contribute to the development of DCM [121, 127]. Circulating lncRNAs predict LV diastolic function and remodelling in patients with T2D [59]. The rapidly growing field of non-coding RNA research will likely provide additional insights into non-coding RNAs and the development of DCM.

#### DNA methylation

DNA methylation involves the transfer of a methyl group to cytosine of CpG dinucleotides in promoter regions to form 5-methylcytosine, which typically represses gene transcription. The expression of genes associated with the development of DCM is regulated by the methylation status of CpG islands, for example SERCA2a [106]. Another example is the expression of liver X receptor-α (LXRα), which is increased in cardiac tissue from T1D rats and regulates the expression of fatty acid metabolism genes. Bisulfite genomic sequencing showed significant differences in the methylation status of the CpG island at the LXRα promoter region [42]. Oxidative stress mediates DNA methylation in T1D hearts, which inhibits DNA synthesis and increases p53-dependent cell death signalling. Oxidative stress-mediated mechanisms involve methylation of the gene encoding the p53-inducible cyclin-dependent kinases (cdks) inhibitor p21^WAF1/CIP1^, which inhibits DNA synthesis and prevents the replication of damaged DNA [140]. These data link epigenetic DNA modifications to the pathogenesis of DCM.

#### Histone modifications

Histones package DNA into structural units called nucleosomes, which are the first level of chromatin organization. Each nucleosome consists of an octameric histone core wrapped in 147 base pairs of DNA. Histone tails are modified by a variety PTMs, including methylation, phosphorylation, ubiquitylation, and acetylation, which regulates gene expression. The acetylation status of histones is a major epigenetic mechanism that is mediated by histone acetyltransferases (HATs) and histone deacetylases (HDACs). HDACs play a critical role in embryonic development, cardiac hypertrophy and heart failure. There is emerging evidence that HDACs are involved in the development of DCM, as indicated by attenuated interstitial fibrosis and apoptosis following HDAC inhibition in T1D mice [41, 223]. Furthermore, histones H2A, H2B and H4 are modified by O-GlcNAcylation [171], thereby directly linking nutrient availability to gene expression.

### Decreased β-adrenergic signalling

Signalling pathways transduced by the IR and β-adrenergic receptors (βARs) mediate divergent and overlapping pathways in the heart. Recent studies revealed a critical crosstalk between insulin and β-adrenergic signalling, which impairs cardiac contractility in T2D [78, 209]. βAR signalling is increased in heart failure. Studies in humans and animal models show that cardiac insulin signalling is preserved or increased in diet-induced obesity, T2D, and heart failure [55, 181, 219]. Pressure overload-induced hypertrophy and heart failure result in hyperinsulinemia and systemic insulin resistance, which accelerate adverse LV remodelling. This effect is attenuated by systemic insulin deficiency or genetic reduction of cardiac IR-transduced signalling by heterozygous cardiomyocyte-specific deletion of the IR [181]. Importantly, large clinical studies have shown that strict insulin treatment of T2D patients increases mortality, despite a reduced incidence of microvascular complications, such as nephropathy and neuropathy [3].

β_1_AR is the predominant βAR receptor subtype expressed in cardiomyocytes, which couples to stimulatory G protein, G_s_. In contrast, β_2_ARs bind to both G_s_ and inhibitory G protein, G_i_. G_s_-mediated signalling induces cyclic adenosine 3′,5′-monophosphate (cAMP)-dependent activation of protein kinase A (PKA) and phosphorylation of phospholamban, which increases myocyte contractility [220]. IR and βARs share G_i_ [188] and G-protein receptor kinase 2 (GRK2) [49, 50, 201] as common downstream effectors, which serve as nodes linking these two signalling pathways. A functional membrane complex consisting of the IR and β_2_AR was also recently discovered [78, 79]. Insulin stimulates translocation of GRK2 to the IR, which contributes to GRK2-mediated phosphorylation of the β_2_AR and enhanced G_i_-mediated signalling. Insulin increases the expression of phosphodiesterase 4D (PDE4D), which antagonizes cAMP activity and decreases PKA phosphorylation, thereby promoting contractile dysfunction [209]. Induction of PDE4D and contractile dysfunction are attenuated in HFD-fed mice following pharmacological inhibition of GRK2 with paroxetine, a FDA-approved selective serotonin reuptake inhibitor. Similar data were obtained following treatment with the β_2_AR blocker Carvedilol [209]. This mechanism provides a potential explanation for the harmful effects of intensive insulin treatment observed in T2D patients, suggesting GRK2 and β_2_AR as potential promising pharmacological targets for the treatment of cardiomyopathy in T2D.

### Increased cell death

Increased apoptotic and necrotic cardiomyocyte death is commonly detected in patients [6, 46, 77] and rodent models of T1D and T2D [10, 22, 96, 103, 173, 232]. Right atrial appendages from T1D and T2D patients subjected to elective coronary artery bypass surgery exhibit increased rates of apoptosis and necrosis, which are exacerbated following simulated ischemia/reperfusion [46]. The proposed mechanisms triggering cell death are increased caspase activation [46], ROS production [96], ER stress [173], activation of death-receptor- and mitochondrion-dependent pro-apoptotic pathways [22], RAAS activation [103], and leptin deficiency, as indicated by decreased apoptosis in ob/ob mice following leptin treatment [10].

## Structural and functional consequences

### Increased fibrosis

In DCM, increased collagen accumulation is observed in perivascular loci, between myofibers, and as replacement fibrosis [161]. Similarly, type III, but not type I or IV collagen deposition, is increased in myocardial biopsies from T2D patients without prior history of hypertension and coronary artery disease [182]. Increased myocardial fibrosis may contribute to diastolic dysfunction in DCM. Serum concentrations of the carboxy-terminal propeptide of procollagen type I (PIP), a marker of myocardial fibrosis, are increased in T2D patients with overt diastolic function, in which lower mitral and tricuspid E/A ratios were detected [97]. Fibrosis is also increased in some animal models of T1D [37, 187, 203, 210] and T2D [138, 232]. The mechanisms responsible for increased fibrosis and connective tissue content include AGE-mediated remodelling of the extracellular matrix (ECM), increased transforming growth factor β (TGFβ)-mediated signalling, increased connective tissue growth factor (CTGF) expression, and decreased expression of MMP-2, resulting in attenuated extracellular matrix degradation [203].

### Diastolic dysfunction

**Fig. 3:**
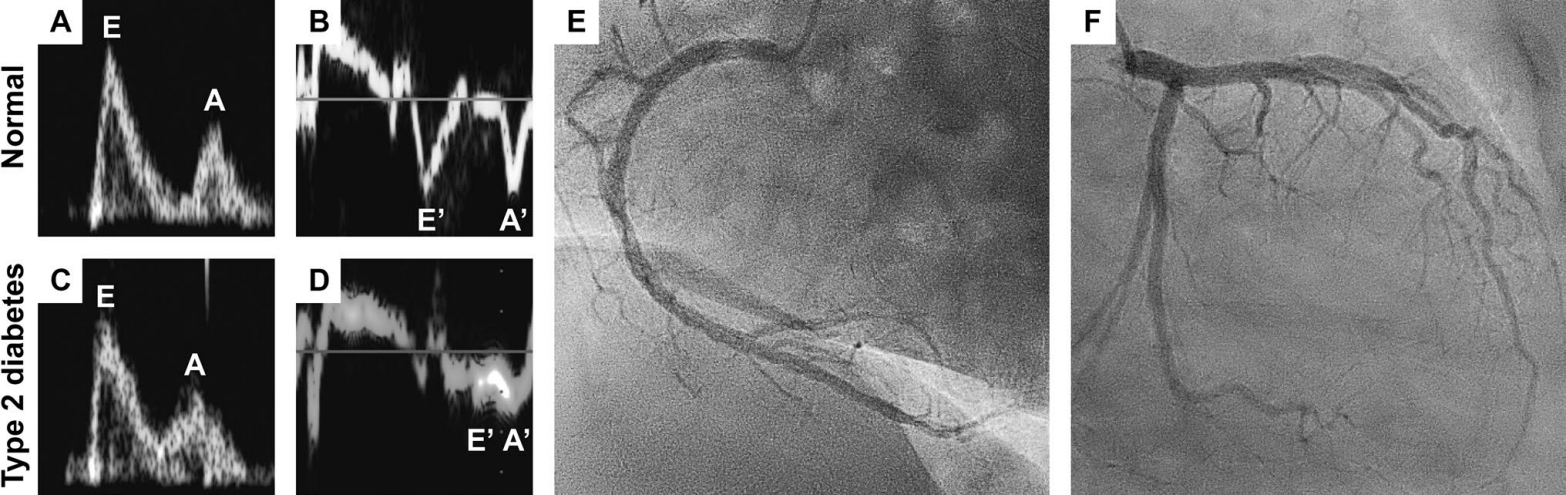
Diastolic dysfunction in the absence of coronary artery disease in a patient with type 2 diabetes. **a** Preserved diastolic function in a normal subject as indicated by the *E*/*A* wave ratio (*E*: peak velocity blood flow in early diastole, *A*: peak velocity blood flow in late diastole caused by atrial contraction), **b** *E*′/*A*′ wave ratio (*E*′: peak mitral annular velocity during early diastolic filling, *A*′: peak mitral annular velocity during late diastolic filling caused by atrial contraction). Diastolic dysfunction in a patient with type 2 diabetes as indicated by **c** an abnormal high (“pseudonormal”) *E*/*A* wave ratio and *E*/*E*’ wave ratio as calculated from the values presented in panels (**c**) and (**d**). Images were adjusted to the same scales. Coronary angiogram of the **e** right coronary artery system and **f** left main coronary artery system from the same patient presented in panels (**c**/**d**) indicating no concomitant coronary artery disease

A key clinical feature of DCM is diastolic dysfunction (see Fig. 3) with preserved ejection fraction (HFpEF), which may precede the later onset of systolic dysfunction (heart failure with reduced ejection fraction, HFrEF). As discussed in the previous sections, various mechanisms contribute to diastolic dysfunction, including AGE, increased fibrosis, and perturbed Ca^2+^ homeostasis. Diastolic dysfunction may be present in as many as 60–75% of diabetic patients, and is even more apparent under conditions of superimposed myocardial ischemic heart disease or hypertension [16, 122, 184]. Furthermore, LV diastolic dysfunction may be present under conditions of insulin resistance even before the onset of T2D, independently of age, blood pressure, and body mass index [75]. After exclusion of patients with coronary artery disease, systolic dysfunction is detected in 24% of T2D patients, as determined by strain analyses and peak systolic velocity measurements [69]. While studies have reported diastolic dysfunction in T1D patients [31, 174, 229], reports on systolic function in T1D are inconsistent, with some studies indicating preserved [168, 174] or increased systolic function [47]. The different results for studies with T1D and T2D patients may be a consequence of the selection of patients and the causative treatment of T1D patients with exogenous insulin, which may normalize the systemic milieu.

Cardiac contractile function has also been extensively investigated in numerous animal models of T1D and T2D. Both diastolic and systolic dysfunction has been observed in some of the models investigated (see Table 1). Importantly, the majority of studies with animal models did not use blood glucose level-normalizing treatments, which further emphasizes the impact of hyperglycaemia and metabolic disturbances on the development of contractile dysfunction.

### Cardiac hypertrophy

Another clinical feature of DCM is LV hypertrophy, especially in T2D. While data from the Framingham Heart Study and the Framingham Offspring Study show an association between diabetes, LV wall thickness and mass in women, but not in men [80], the Strong Heart Study conducted in Native Americans reports increased LV mass and wall thickness in both men and women [62]. Data from the Strong Heart Study suggest that LV hypertrophy increases the risk of future heart failure, especially in the context of co-existing hypertension [17]. LV hypertrophy is not observed in patients with impaired fasting glucose [162], indicating that LV hypertrophy might result from hyperglycaemia and other metabolic changes associated with longer existing diabetes. Proposed mechanisms contributing to LV hypertrophy are hyperactivation of the insulin signalling cascade in obese and T2D patients [55, 108] and increased levels of circulating pro-inflammatory cytokines. In contrast, most studies do not report myocardial hypertrophy in T1D patients [47, 168, 174] and animal models of T1D (see Table 1). Similarly, genetic deletion of the insulin receptor decreases cardiac size [14]. These studies further highlight the impact of insulin as a growth factor and hyperinsulinemia as a pathomechanism for LV hypertrophy in obesity and T2D.

## Summary and conclusions

Various pathomechanisms contribute to the pathogenesis of DCM. Rodent models are essential tools to decipher these mechanisms and mimic perturbations observed in T1D and T2D patients. Despite specific limitations of the models generated, transgenic mice are indispensable for mechanistic studies that provide mechanistic insight into the pathogenesis of DCM. Different treatment strategies have been tested in patients with diabetes mellitus and heart failure. These studies indicate that diabetic patients benefit from standard heart failure treatment. However, previous studies also suggest that selected diabetes mellitus treatment regimens may have adverse effects on cardiac function and increase heart failure hospitalization [130, 164]. These observations also emphasize the need for additional studies to gain further mechanistic insight. Recent advancements in genome editing will result in the generation of novel models in the near future. These models will aid our understanding of the pathophysiology of DCM and hopefully accelerate the development of new therapeutic strategies for this rapidly expanding form of heart disease.
